# Learning and change in a dual lexicon model of speech production

**DOI:** 10.3389/fnhum.2023.893785

**Published:** 2023-02-15

**Authors:** Maya Davis, Melissa A. Redford

**Affiliations:** Department of Linguistics, University of Oregon, Eugene, OR, United States

**Keywords:** computational model, development, exemplar theory, schema theory, speech motor plan

## Abstract

Speech motor processes and phonological forms influence one another because speech and language are acquired and used together. This hypothesis underpins the Computational Core (CC) model, which provides a framework for understanding the limitations of perceptually-driven changes to production. The model assumes a lexicon of motor and perceptual wordforms linked to concepts and whole-word production based on these forms. Motor wordforms are built up with speech practice. Perceptual wordforms encode ambient language patterns in detail. Speech production is the integration of the two forms. Integration results in an output trajectory through perceptual-motor space that guides articulation. Assuming successful communication of the intended concept, the output trajectory is incorporated into the existing motor wordform for that concept. Novel word production exploits existing motor wordforms to define a perceptually-acceptable path through motor space that is further modified by the perceptual wordform during integration. Simulation results show that, by preserving a distinction between motor and perceptual wordforms in the lexicon, the CC model can account for practice-based changes in the production of known words and for the effect of expressive vocabulary size on production accuracy of novel words.

## Introduction

How do we produce an unfamiliar word that we have just heard? One answer is that we hear and encode the word as a sequence of phonemes; when the sequence is activated for production, the phonetic aspect is filled in, syllable structure is imposed, and the corresponding motor programs are selected and executed (Levelt, 1989; Levelt et al., 1999; Guenther, 2016). But, if our production of the unfamiliar word is inaccurate, how exactly do we improve on it over time? The Computational Core (CC) model presented in this paper was built to address this question and others that arise from the developmental problem of learning and change in production— learning and change that occurs across the lifespan.

One approach to the problem of learning and change in production is to assume both perceptual representations linked to phonemes and online control over execution (e.g., Houde and Nagarajan, 2011; Parrell et al., 2019). Under these assumptions, predictive control can be used to adjust a planned articulation that will miss the acoustic goal linked to a phoneme (Niziolek et al., 2013). But what if the unfamiliar word that a speaker attempts makes use of familiar phonemes linked to unfamiliar sounds arranged according to an unfamiliar timing pattern? The standard approach to this problem, encountered in adult second language learning, is to assume perceptual learning at the level of the acoustic categories that define speech motor goals (Flege, 1995; Samuel and Kraljic, 2009; Holt and Lotto, 2010; Flege and Bohn, 2021). Such learning could induce change in production based on online control. Yet, studies on second language acquisition indicate that accurate perceptual learning does not result in production accuracy (Nagle and Baese-Berk, 2022), especially if the newly learned acoustic category cannot be mapped onto a speaker's prior production experience (Nielsen, 2011; Nagle, 2018). Despite learning, changes in production accuracy are constrained.

Also, even if an unfamiliar sound can be attained based on perceptual learning, how is an unfamiliar timing pattern achieved? Native-like production of relative timing patterns within a word are acquired early by first language speakers, but not nearly as easily—if ever—by adult second language speakers (e.g., Redford and Oh, 2017). The question of how relative timing patterns are acquired is especially difficult to address within a framework where word production and perception are mediated by phonemes. An alternative approach is to assume that learning is instead mediated by wordform representations. For example, the detailed acoustic-perceptual wordform representations of exemplar-based theories (Johnson, 1997, 2006; Pierrehumbert, 2002; Smith and Hawkins, 2012) necessarily include time-varying information about acoustic goals that could be referenced during execution. Predictive control could be used to adjust planned articulations accordingly, which would result in changes to production. But, if accurate production of unfamiliar words with unfamiliar sounds and timing patterns can be attained simply with reference to whole-word perceptual representations, then why is the correlation between perception and production in second language acquisition so far from perfect? Put another way: What constrains production during learning? Relatedly, why does production accuracy, measured against perceptual input, appear to plateau in adult second language speakers?

The typical explanation for constrained production accuracy in second language speech is that unfamiliar words are not directly read off from perceptual representations; rather, they are filtered through a speaker's phonology (Major, 1998, 2001). In exemplar-based theories, the phonology is language-specific knowledge about phonemes, phonotactics, and other suprasegmental patterns abstracted from across the perceptual wordforms of the lexicon (Bybee, 2002; Pierrehumbert, 2003). When these abstractions are stored (“labeled”) separately from the lexicon, an exemplar-based model of production makes assumptions similar to phoneme-driven models of production (see, e.g., Pierrehumbert, 2001; Wedel, 2006); that is, it assumes acoustic goals linked to phonemes and so it assumes phoneme-guided production. Given that time-varying information must also be learned and implemented by the motor system to effect change in production, this type of model is unsatisfactory. The CC model presents a word-based alternative to the phoneme-driven model of production. The goal of the model is to account for perceptually-driven learning and change in production and for the constraints on said change.

The CC model addresses learning and change from a developmental perspective. This perspective is adopted because (a) the problem of learning and change is especially acute in early language development, and (b) the adult's production system emerges from the child's and so should be derived from it. The latter reason constitutes a working hypothesis that has led us to propose a developmentally sensitive theory of speech production (Redford, 2015, 2019)—a framework for understanding the evolution of speech production across the lifespan. The CC model details an important piece of the theory: the idea that speech motor processes and phonological forms influence one another because speech and language are acquired together. The model instantiation of this idea captures language-specific limits on perceptually-driven motor learning and change in production.

## Background to the CC model

The CC model assumes a dual lexicon. More specifically, it assumes a lexicon comprised of separate perceptual and motor wordforms that are jointly linked to shared concepts. The CC model also assumes whole-word production. These assumptions are motivated by our developmental perspective. Both extend specific ideas from child phonology to provide the basis for a developmentally sensitive account of adult production.

The shapes of children's first words deviate markedly from adult wordforms. Work in child phonology shows that these deviations are idiosyncratic. For example, one child will say [bɑbɑ] for *bottle* (Velleman, 1998; cited in Velleman and Vihman, 2002, p. 20) while another says [bɑdi] (Vihman, 2014, p. 80) and a third says [papm:] (Jaeger, 1997; Vihman and Croft, 2007, p. 702). The idiosyncratic productions of single words are associated with child-specific systematicities across multiple words. For example, the 18-month-old who says [pɑpm:] for “bottle” replaces voiced stops with voiceless ones in “baby” and “byebye,” rendering these as [peipi] and [(pə)pa:i], respectively; she also produces word-final nasals in other words where they are not required (e.g., [kʌkŋ] for “cracker” and [takŋ] for “doggie”; see Table 9 in Vihman and Croft, 2007, p. 702). In general, children's deviations from adult-like wordforms are interpreted to suggest strong motor constraints on first word production (Menn, 1983; Nittrouer et al., 1989; McCune and Vihman, 2001; Davis et al., 2002). Ferguson and Farwell (1975) proposed that individual children overcome these constraints by applying their favored sound patterns to best approximate whole word targets, resulting in systematic patterns of individual difference in production. McCune and Vihman (2001) went further to specify that a child's favored patterns are selected from among their vocal motor schemes that are established with vocal-motor practice during the pre-speech period. Redford (2015) combined this idea with the ideas of generalized motor programs from schema theory (see Schmidt, 1975, 2003) and gestural scores from Articulatory Phonology (Browman and Goldstein, 1986, 1992) to propose that, even beyond the first word period, the child continues to rely on established motor representations to guide production and that this reliance continues on through adulthood.

**Figure 1:**
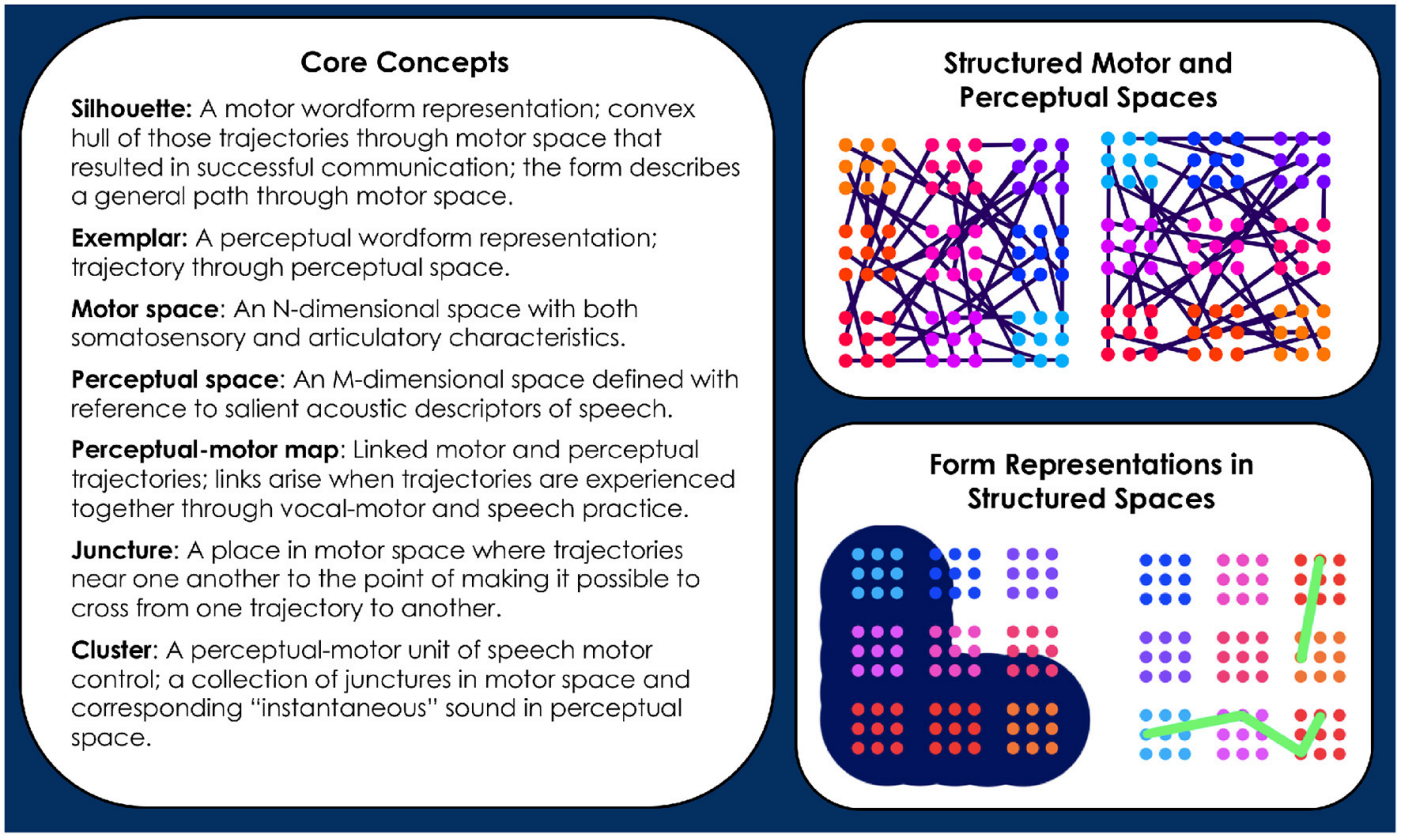
Informal definitions of Core concepts are provided (see text for detail). The illustrations to the right of the definitions depict several of the concepts. The top right panel depicts 2-dimensional motor **(left)** and perceptual **(right)** spaces that have already been structured by the trajectory crossings that occur with vocal-motor exploration and speech practice. Junctures are represented as dots, clusters as groups of identically colored dots. Each cluster of a particular color in motor space corresponds to one of the same color in perceptual space. Links between the motor and perceptual spaces are assumed but not shown. The bottom right panel depicts a silhouette **(left)** and an exemplar **(right)** in relation to the motor and perceptual spaces, respectively. The depiction of the silhouette highlights the idea that it describes a broad path through motor space. The depiction of an exemplar highlights its status as a specific trajectory through perceptual space. The distinct layouts of clusters in the simplified motor and perceptual spaces illustrates that these spaces have different topologies.

In Redford (2015), the motor representations that guide production were defined as temporally-structured memories built up from motor traces associated with the successful communication of concepts. They are first established when communication of a new concept is first attempted. Of course, this first attempt requires that the child also have stored a perceptual representation of the wordform that denotes a concept. This representation serves as the goal for production. Its presence in the lexicon allows for developmental change in the direction of the adult form (Redford, 2019). But, with a hypothesis of whole-word production, comes the problem of how to explain the emergence of segment-like control over speech articulation. Davis and Redford (2019) proposed the Core model to address this problem. In brief, Core demonstrated that segment-like control could emerge under the assumption of whole-word production with practice-based structuring of the perceptual-motor map. This specific solution to the problem entailed formalizing a number of concepts that are also central to the CC model. Figure 1 itemizes and illustrates these concepts for quick reference. More complete descriptions of the concepts follow.

### Core concepts

The CC model assumes that motor wordforms are established with reference to perceptual wordforms and that, once established, the motor and perceptual forms are integrated during production (Redford, 2019). We first formalized this hypothesis in the Core model (Davis and Redford, 2019). In so doing, we defined a lexicon of perceptual and motor wordforms with respect to a *perceptual space* and a *motor space*.

The perceptual space is the set of all possible instantaneous sounds, along with a distance metric and subsequent topology. The motor space is the set of all possible articulatory configurations, along with a distance metric and subsequent topology. The perceptual and motor spaces are grounded in the acoustic and articulatory dimensions of speech. This grounding is assumed but not defined in the CC model. In Davis and Redford (2019) the dimensions were as follows. A point in perceptual space was represented by coordinates measuring sound periodicity, Bark-transformed formant values, the spectral center of gravity, the width of the spectral peak, and the time derivatives of the formant and other spectral measures, as well as the time derivative of amplitude. A point in motor space was represented by coordinates measuring glottal width, the cross-sectional areas of 8 regions of the vocal tract from lips to larynx, the time derivatives of each of the cross-sectional areas, velum height, the time derivative of velum height, and the direction and force of the opening/closing movement of the jaw. Euclidean distance metrics were used to calculate the relationship between points in these spaces.

The perceptual wordform, defined with respect to perceptual space, is called an *exemplar*. The label indicates our embrace of exemplar-based accounts of phonology, sociolinguistic knowledge, and perceptual learning. None of these topics are explicitly addressed here. Instead, the exemplar is merely a precise whole-word perceptual representation. It is a function that takes a moment in time as an input and gives as an output a point in perceptual space. Such a function describes a trajectory through perceptual space; it is called an exemplar only when linked to a concept.

The motor wordform, defined with respect to motor space, is called a *silhouette*. It is a temporally-structured memory of the movements needed to achieve a wordform that communicates a concept. It is built up over time whenever its concept is successfully communicated. It is most analogous to the idea of a generalized motor program (GMP) for skilled action (Schmidt, 1975, 2003), except that it is a more specific representation than the GMP. Unlike a GMP, a silhouette is effector-dependent: it is defined along dimensions determined by possible movements of the speech articulators.

**Figure 2:**
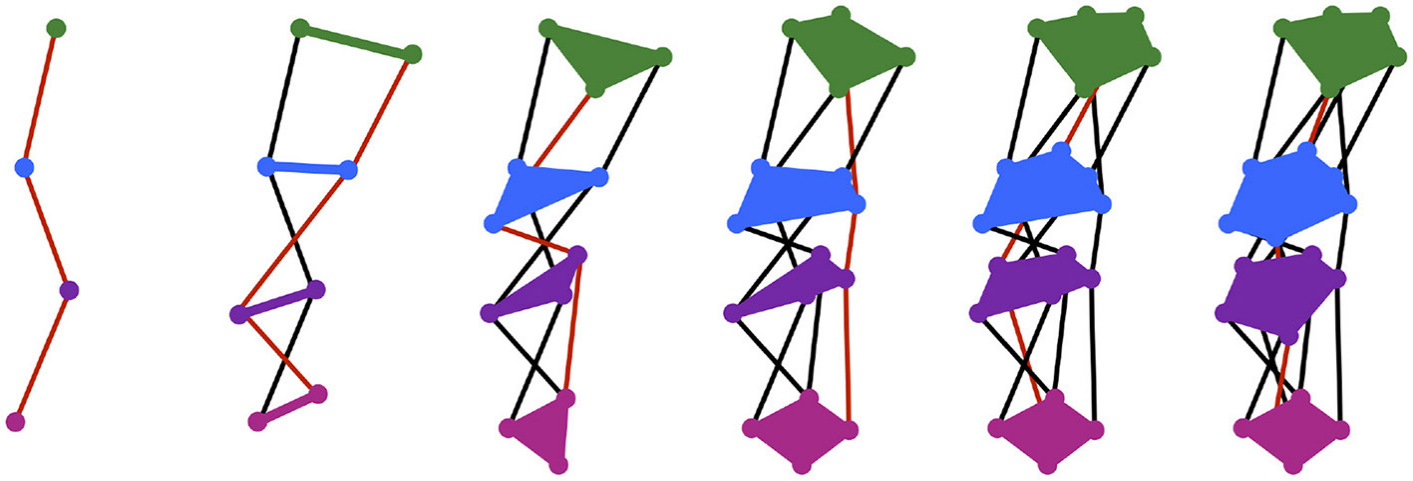
Each panel shows the silhouette expanded from the previous panel to include an additional motor trajectory, whose path is shown in red. The regions of the silhouette at four time steps are drawn—the region at the first time is shown in green, at the second time in blue, at the third time in purple, and at the fourth time in pink—but theoretically infinitely many regions exist along the whole length of the silhouette. Silhouette expansion is a continual process. Motor trajectory traces are added whenever communication succeeds.

In first-word production, exemplars are purely exogenous representations. Silhouettes are endogenous representations that begin to emerge when the infant first successfully communicates a concept *C* by targeting the exemplar, *e*_*C*_. The silhouette for the concept, SIL_*C*_, is a function that takes a point in time as an input, and gives as an output a region in motor space that describes a general vocal tract configuration to be targeted by the motor system at that time. As with the exemplar, the subscript *C* denotes the silhouette's link to the concept *C*. Each time *C* is successfully communicated, SIL_*C*_ expands to include a trace of the motor trajectory, *m*, that was executed. More specifically, for each time *t*, the region SIL_*C*_(*t*) expands the smallest amount possible such that (1) the new region also includes *m*(*t*) (as well as the old region) and (2) the new region is convex. In the CC model, new and old regions are also weighted over time with the addition of new traces representing successful communication of *C*, which effectively skews the silhouette in the direction of the most frequently used motor trajectories. An illustration of motor silhouette expansion is shown in Figure 2. Silhouette weighting is not shown; it is instead described at length later in this paper.

First word production is the effective communication of a novel concept *C* that has been learned along with *e*_*C*_ from the ambient language. The infant first achieves communication of *C* through a matching and selection process that leverages motor trajectories established through babbling and other vocal-motor exploration. Because the trajectories in motor space are self-produced, they are automatically linked to perceptual trajectories in perceptual space. The linked motor and perceptual trajectories make up the *perceptual-motor map* that is exploited during the matching and selection process used to attempt a new word. This process computes the distance in perceptual space between an exemplar and the perceptual aspect of established motor trajectories through motor space. The computation allows for the combination of multiple established trajectories, one after another in time, to best approximate the intended exemplar. Along the way, the matching and selection process structures the perceptual-motor map by creating *junctures*, which are motor points at which the speaker shifts from one established trajectory to another nearby one.

Even during the initial stages of vocal-motor exploration, very specific regions of motor space are passed over multiple times in a variety of trajectories (e.g., the [ɑ] region in babbled utterances “bɑbɑ” and “dɑdɑ”). In Davis and Redford (2019), we proposed that frequently traversed regions in motor space become populated with junctures through the matching and selection process during the first word stage of development. The specific suggestion was that children create junctures when they combine chunks of previously experienced perceptually-linked motor trajectories in their first word attempts. For example, a child will first link the perceptual and motor spaces of speech during the pre-linguistic period, including with trajectories such as “bɑbɑ" and “dɑdɑ" produced during the babbling phase. When this child first attempts the word “bottle” they may seek to match its perceptual form by leveraging the “bɑbɑ" or “dɑdɑ" trajectory. They may even combine these trajectories to produce “bɑdɑ" by following the (motor) path for “bɑbɑ" and then transitioning to the path for “dɑdɑ" where the two trajectories (nearly) meet in the [ɑ] region of motor space. If the resulting “bɑdɑ” trajectory contributes to communicative success (e.g., receiving the requested bottle), then the motor trace of the “bɑdɑ" trajectory is stored with a link to the concept “bottle.” This trace provides the first outline for the silhouette associated with that concept (see Figure 2).

As junctures proliferate with vocal-motor practice and vocabulary expansion, they are grouped together based on their proximity to one another in motor space. These groupings are *clusters*. A cluster designates a specific region in motor space that is crossed over and over again while achieving similar sounds within various words. Over developmental time, clusters begin to serve as perceptual-motor units of control. They can be targeted quasi-independently because they designate regions within motor space that many trajectories go through, allowing the speaker to target the region from many other locations within the space. At a higher level of abstraction, clusters represent turning points in motor trajectories. These turning points can be conceived of as linguistically-significant vocal tract constrictions—something similar to “gestures” in Articulatory Phonology (Browman and Goldstein, 1986, 1992), albeit with context-dependent timing that is defined by the trajectory leading into and out of the turning point. In perceptual space, clusters represent a quasi-static acoustic goal associated with a particular articulatory configuration—such as the sound that we might associate with a segment (e.g., [ɑ]) or with a critical feature (e.g., the silence of stop closure). Although it is possible to associate clusters with gestural or featural descriptions of the phonology, we stress that they are simply units of speech motor control. Clusters only exist at the level of the perceptual-motor map. They do not necessarily create meaning contrasts. They emerge from and remain embedded in a well-defined perceptual-motor context.

Having introduced the Core concepts of perceptual and motor spaces, exemplars, silhouettes, the perceptual-motor map, junctures, and clusters, we are ready to describe the CC model. This model picks up after the first-word stage where the mathematical Core model leaves off.

## Architecture of the CC model

In Davis and Redford (2019), we modeled the first-word stage of spoken language development and its structuring effects on the perceptual-motor map. In this paper, we model word production at a later stage in development; a stage when the perceptual-motor map has already been structured with speech practice and so is already discretized into clusters. This new focus entails making explicit the relationship between wordform representations and the perceptual-motor map. This relationship is critical to the perceptual-motor integration of wordforms that is at the heart of speech production in the theory.

The silhouette and exemplar activate clusters in motor and perceptual space, respectively. In the CC model, sequential information is preserved by the silhouette with the time-varying activation of clusters in motor space.^1^ By contrast, the exemplar activates all its clusters at the same time in perceptual space. The time-varying activation of clusters in motor space is consistent with the ecological–dynamic hypothesis that phonological representations incorporate time-varying (i.e., dynamic) information (Fowler, 1980; Browman and Goldstein, 1986, 1992). The simultaneous activation of clusters in perceptual space is consistent with the structural hypothesis that paradigmatic relations are more important than syntagmatic ones when acoustic-auditory categories serve as speech motor goals (Diehl and Lindblom, 2004; Flemming, 2004). Very importantly, the different activation patterns ensure unique motor and perceptual contributions to wordform integration. The silhouette-driven activation pattern highlights context-dependent constraints on articulation. The exemplar-driven activation pattern highlights the goal of attaining (more) context-independent sounds in articulation. The different activation patterns and their specific consequences are inspired by Lindblom's (1990) H&H theory of production. Lindblom proposes that speakers have two modes of production, a hypo mode and a hyper mode, that serve as ends of a speaking style continuum. The hypo mode results in highly coarticulated speech. The hyper mode results in more context-independent attainment of acoustic goals. The CC model reflects these extreme modes in its different activation patterns of motor and perceptual space.^2^

The silhouette and exemplar are integrated with cluster activation. More specifically, the activation pattern across clusters in motor space and the activation pattern across clusters in perceptual space are combined and used to determine a trajectory through the perceptual-motor map that guides speech movement. Look-ahead and look-back windows specify the extent to which information about the combined activation pattern in the future and/or past is incorporated into the current activation pattern. At any given time, the integration process thus results in the differential activation of multiple clusters. As clusters represent perceptual-motor units that are both spatial targets and perceptual goals, the simultaneous activation of several of these at once means that articulation represents a compromise between competing targets/goals.

Overall, the CC model claim is one of real-time speech motor planning and execution. Speech motor control is not modeled but the planning process remains compatible with current models (e.g., Houde and Nagarajan, 2011; Guenther, 2016; Parrell et al., 2019). In what follows, the production process from cluster activation to perceptual-motor integration to the computation of the (perceptual-)motor output trajectory is formally described. We would point those interested in further detail to the source code, which is available on GitHub (https://github.com/mayaekd/core).

### Cluster activation

Let *C* be a word-sized concept. The speech plan for *C* is the activation pattern of clusters in the perceptual-motor map that results from the selection of the silhouette that corresponds to *C*, SIL_*C*_, and an exemplar, *e*_*C*_, chosen from among the set of exemplars associated with *C*. The perceptual-motor map itself contains many clusters: CLUSTER_1_, CLUSTER_2_, … , CLUSTER_*n*_. Each of these is made up of some number of junctures; assume CLUSTER_*i*_ is made up of JUNCTURE_*i*,1_, JUNCTURE_*i*,2_, … , JUNCTURE_*i*,*mi*_. The silhouette, SIL_*C*_, activates clusters in motor space while the exemplar, *e*_*C*_, activates clusters in perceptual space. For the reasons explained in the preceding section, the activation of clusters in motor space varies across time; the activation of clusters in perceptual space is simultaneous. The details of the activation patterns are as follows.

#### Activation in motor space

First, the silhouette activates the region in motor space corresponding to the first step on the time interval. At the next time step, it activates the next corresponding region. At the one after that, the next region is activated, and so on until the path through motor space associated with the entire silhouette has been traversed.

**Figure 3:**
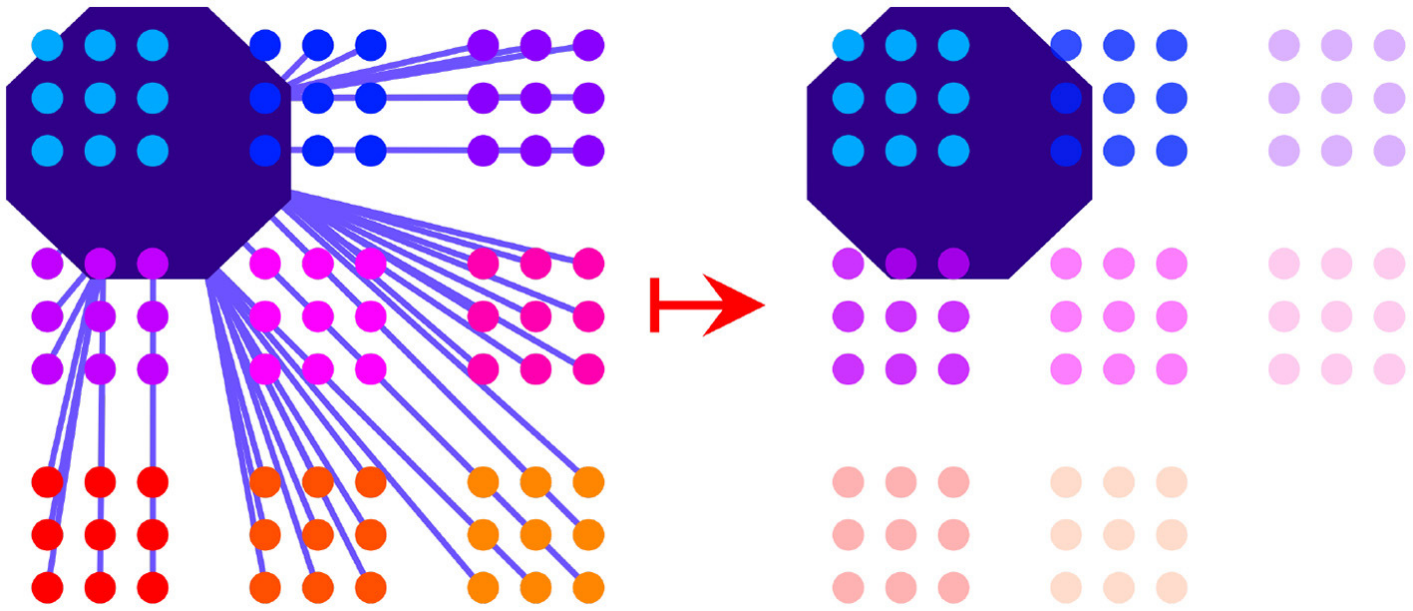
The activation process in motor space is shown. The region of the silhouette at a particular time step activates junctures that are overlapping with the region or less than a certain distance away from it (distances shown by purple lines, **left**). Activation spreads evenly within a cluster. Activation levels are determined by the distances of the junctures to the silhouette region. Activation strength of clusters is depicted by the relative transparency-opacity of the clusters **(right)**.


When a region in motor space is activated, the activation immediately spreads across junctures that are inside that region or within a certain distance of that region. Juncture activation spreads evenly within the bounds of each cluster. This means that clusters are activated as units within motor space. Clusters that are further away from the region that is highlighted by a silhouette at a particular time step will be less activated than those that are closer to the region or are in the region itself, as depicted in Figure 3. More precisely, the motor activation at time *t* of CLUSTER_*i*_ is defined to be the average of the motor activation of every juncture in that cluster:

$$\begin{array}{l} {\text{M}\text{OTOR}\text{A}\text{CTIVATION}}_{t}\left({\text{C}\text{LUSTER}}_{i}\right)=\frac{1}{m_{i}}\overset{m_{i}}{\underset{j=1}{\sum }}{\text{M}\text{OTOR}\text{A}\text{CTIVATION}}_{t}\\ \;({\text{J}\text{UNCTURE}}_{i,j}) \end{array}$$

Where the motor activation of JUNCTURE_*i,j*_ is defined to be the highest when JUNCTURE_*i,j*_ is contained in SIL_*C*_(*t*) and to fall off linearly as the distance between JUNCTURE_*i, j*_ and SIL_*C*_(*t*) increases, bottoming out at zero:


$$\begin{array}{l} {\text{M}\text{OTOR}\text{A}\text{CTIVATION}}_{t}\left({\text{J}\text{UNCTURE}}_{i,j}\right)=\text{H}\text{IGHEST}\text{A}\text{CTIVATION}\text{M}\text{OTOR}\\ \;-\left(\text{D}\text{ROP}\text{O}\text{FF}\text{S}\text{LOPE}\text{M}\text{OTOR}\times \text{D}\text{ISTANCE}\left({\text{SIL}}_{C}\left(t\right),{\text{J}\text{UNCTURE}}_{i,j}\right)\right) \end{array}$$

We generally set


HIGHESTACTIVATIONMOTOR=1

and


DROPOFFSLOPEMOTOR=0.1.

Although we refer here to the motor activations of the junctures, note that this should be thought of as an initial theoretical state of the cluster that is quickly changed once the activation spreads within a cluster.

#### Activation in perceptual space

Although the exemplar is also a function on a time interval, its set of points activate nearby junctures in perceptual space all at once when the exemplar is selected. Similar to juncture activation in motor space, activation spreads outwards from points along the exemplar trajectory; activation also decreases in strength with distance from the exemplar trajectory, and the activation is averaged across the points in the exemplar. Again, activation spreads so that all junctures within a particular cluster receive the same activation. For an exemplar consisting of points *p*_1_, …, *p*_*r*_, and a cluster CLUSTER_*i*_ consisting of junctures {JUNCTURE_*i*,1_, …,JUNCTURE_*i*,*mi*_}, we can write


$$\begin{array}{l} {}[\text{E}\text{XEMPLAR}\text{A}\text{CTIVATION}(\text{C}{\text{LUSTER}}_{i})=\frac{1}{m_{i}}\sum_{j=1}^{m_{i}}\;\\ \;\text{E}\text{XEMPLAR}\text{A}\text{CTIVATION}(\text{J}{\text{UNCTURE}}_{i,j}) \end{array}$$

where


$$\begin{array}{l} \text{E}\text{XEMPLAR}\text{A}\text{CTIVATION}(\text{J}{\text{UNCTURE}}_{i,j})=\frac{1}{r}\overset{r}{\underset{k=1}{\sum }}\\ \;(\text{H}\text{IGHEST}\text{A}\text{CTIVATION}\text{P}\text{ERCEPTUAL}\\ \;-(\text{D}\text{ROP}\text{O}\text{FF}\text{S}\text{LOPE}\text{P}\text{ERCEPTUAL}\\ \;\times \text{D}\text{ISTANCE}(p_{k},\text{J}{\text{UNCTURE}}_{i,j}))). \end{array}$$

Like in the motor case, we generally set


HIGHESTACTIVATIONPERCEPTUAL=1

and


DROPOFFSLOPEPERCEPTUAL=0.1

### Perceptual-motor integration

The silhouette and exemplar are integrated as follows to produce speech output. First, the combined activation pattern across the motor and perceptual spaces is computed. This pattern consists of activation that varies by time and by cluster, and is determined by the following equation for the activation at time *t* of cluster CLUSTER_*i*_:


$$\begin{array}{l} \text{A}{\text{CTIVATION}}_{t}(\text{C}{\text{LUSTER}}_{i})=({\text{M}\text{OTOR}\text{A}\text{CTIVATION}}_{t}({\text{C}\text{LUSTER}}_{i})\\ {\;\times \text{E}\text{XEMPLAR}\text{A}\text{CTIVATION}({\text{C}\text{LUSTER}}_{i}))}^{\frac{1}{2}} \end{array}$$

We take the geometric mean (multiplicative mean) of the two activations rather than the arithmetic mean (additive mean) in order to determine the combined activation of a cluster in a way that ensures the correct sequencing of articulatory movements. The geometric mean functions as an AND gate rather than as an OR gate to activation—if the activation of a cluster in either motor or perceptual space is zero, then the combined activation of that cluster is zero. Multiple clusters may compete to influence articulation, but competing clusters should all be within some limited distance of the region specified by the silhouette at that moment in time. If they are not, they should not influence articulation at all. Although the same constraint applies to both spaces, the constraint from motor space is more important. By ensuring that zero activation of a cluster in motor space cannot be overridden by some activation of the cluster in perceptual space, we are ensuring that activation from parts of the exemplar trajectory not relevant to the current time do not have an overwhelming influence on the output trajectory at that time.

The activation values of the cluster vary over time. When activation is computed for a specific time *t*, this yields a set of values *a*_*i*_(*t*), for *i* = 1, …, *n*, where *a*_*i*_(*t*) is the activation of CLUSTER_*i*_. The CC model assumes that the motor system works out a compromise among the various clusters. In the model, the estimated outcome of this compromise at time *t* is computed as the weighted average of cluster locations in motor space, with the weights being the activations of the clusters at time *t*. That is, the estimated motor coordinate list, ESTMOTOR(*t*), is defined as:


$$\begin{array}{l} \text{E}\text{ST}\text{M}\text{OTOR}\left(t\right)=\frac{\sum_{i=1}^{n}a_{i}\left(t\right)\times \text{M}\text{OTOR}\text{C}\text{ENTER}\left({\text{C}\text{LUSTER}}_{i}\right)}{\sum_{i=1}^{n}a_{i}\left(t\right)}, \end{array}$$

Where MOTORCENTER(CLUSTER_*i*_) is the motoric center of CLUSTER_*i*_, which could be defined multiple ways, but which we choose to define as the average of all the junctures' motor locations.

**Figure 4:**
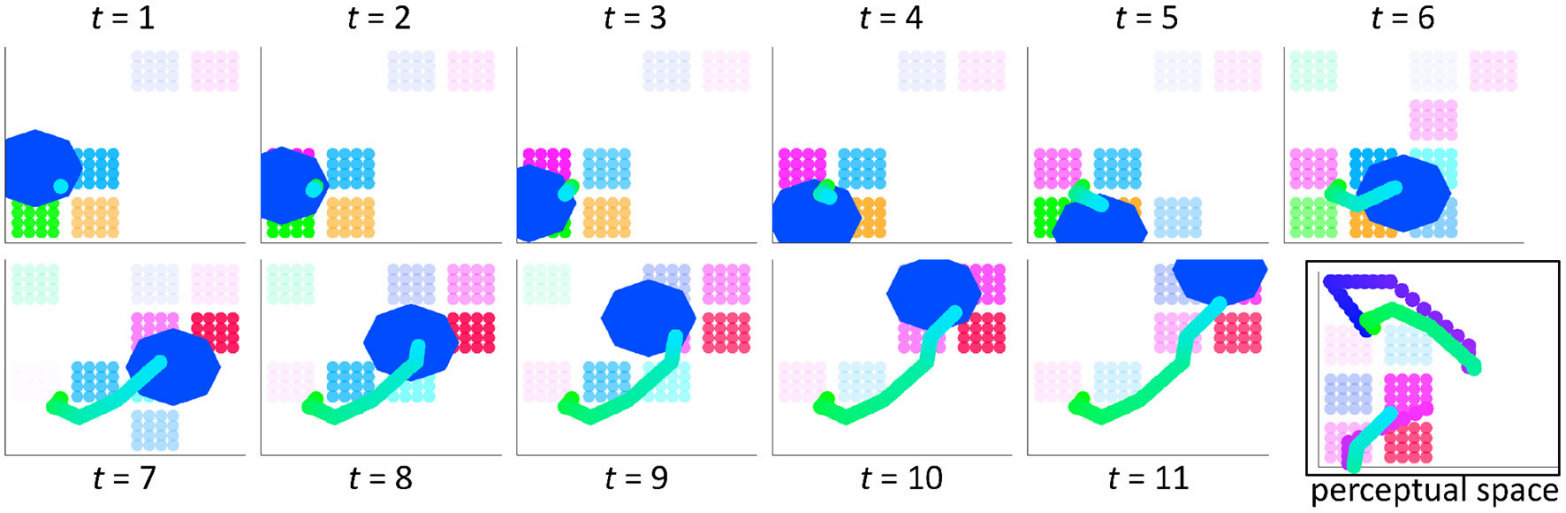
The perceptual-motor integration of wordforms results in an output trajectory through the perceptual and motor spaces, which are linked *via* clusters. Activation strength due to the silhouette and exemplar is depicted by the relative transparency/opacity of the clusters. These clusters are shown here in motor space for a silhouette that is 11 time steps in length. The region of the silhouette (blue octagon) is shown at each time step in this space. The resulting trajectory incorporates directional information (line shading from green to cyan blue). The trajectory is also shown in perceptual space, with the exemplar trajectory (dots shading from blue to pink). It is discontinuous in perceptual space because the topology of this space is different from that of motor space.

When computed for each time step determined by the silhouette, the result of integration is an output trajectory through motor space that reflects the influences from perceptual space due to the exemplar. Figure 4 provides an example of the integration process over 11 time steps (*t* = 11). The combined motor and perceptual activation pattern is shown in motor space, where relative activation is depicted by the relative opacity of the clusters. The trajectory (whose direction is light green to light blue) moves through motor space over time, mainly within the path described by the silhouette. This silhouette path is shown by the region in motor space (the royal blue octagon) that is highlighted at each time step. The full output trajectory for the selected silhouette–exemplar pair is shown at time step 11 in motor space. It is also shown in perceptual space along with the exemplar trajectory. It is represented as a discontinuous trajectory in perceptual space to illustrate that this space has a different topology than motor space and because true discontinuities exist in perceptual space but never in motor space.

Finally, a reminder that not every path through motor space is physically possible because the dimensions of this space are not (usually) independent of one another (e.g., the cross-sectional areas of 8 regions of the vocal tract from lips to larynx and the time derivatives of each of these cross-sectional areas). That said, the CC model assumes a perceptual-motor map that has been structured by experience. Under this assumption, there are a high number of paths that exist between clusters. The path that the motor system chooses to follow is estimated based on the linear combination of cluster weighting. The output trajectory that results could be predicted internally or it could be the trace of movement that has happened. Either way, the output trajectory is a result of cluster activations that are commands to the motor system; it is not itself a control structure.

## Learning and change in production

In the Core/CC model framework, an activated exemplar represents the perceptual goal of speech production. The jointly-activated silhouette constrains goal achievement by biasing movement toward familiar paths through motor space. In first and second language acquisition, these familiar paths are likely to diverge very substantially from the perceptual goal. Over time, path divergence narrows and production accuracy improves. This happens in one of two ways: (1) *via* change in the structure of the perceptual-motor map; (2) *via* change in the shape of existing silhouettes. The Core model addressed the former type of learning; the CC model captures the latter.

### Practice-driven change

Recall that silhouettes are only established after the perceptual-motor map is at least partially structured through prelinguistic speech practice. First word production is based on the perceptual matching and selection process that was described under the Core Concepts section. This process gives rise to the first silhouettes. Once enough silhouettes have been established, speech production is fast and automatic because it is largely driven by silhouette–exemplar pairs that are activated when concepts are selected for communication. The repository of concepts with associated silhouette–exemplar pairs is the expressive vocabulary. It is about half the size of the speaker's overall vocabulary (Brysbaert et al., 2016). The other half is the receptive-only vocabulary. It includes only concept-associated exemplars that the speaker may choose to target at some point.

Production that is guided by the expressive vocabulary will entrench structure at the level of the perceptual-motor map because it constrains production to established motor paths. Accordingly, it will also slow the rate at which speech production patterns change. Some deviation from established paths is possible with the expansion of a silhouette due to random noise.^3^ But, in general, the perceptual-motor integration of wordforms greatly reduces the exploration of new regions in motor space. Also, it is only with a return to a matching and selection process that new junctures and clusters can be generated (see Core Concepts). This means that practice-based changes to speech are initially more likely to occur at the level of wordform representation than at the level of the perceptual-motor map once an expressive vocabulary of a certain size is established. In the CC model, changes to the wordform occurs because practice results in silhouettes with weighted regions. These weighted regions encode frequency information and shift the silhouette in the direction of frequently used output trajectories that meet with communicative success. The details of the weighting algorithm are as follows.

#### Weighted silhouettes

Recall that the silhouette highlights time-varying regions of motor space. The highlighted region is computed as the convex hull of the points associated with previously experienced trajectories (see Davis and Redford, 2019; Sections 2.5.2, 2.5.3). In the CC model, the convex hull is partitioned into simplices (*n*-dimensional “triangles"), each of which are assigned a weight. This means that, at each time, the highlighted region in motor space, returned by the function that is the silhouette, is a weighted homogenous simplicial complex. More specifically, let SIL_*C,n*_ be the silhouette for concept *C* at a particular time in development, denoted by *n*. Assume the current silhouette is *T* (relative) time units long, and let *k* be a sufficiently large number. Then SIL_*C,n*_ is defined to be a function with domain [0, *T*] that takes an input of a particular time and gives an output of the weighted region corresponding to that time in the form of a weighted simiplicial complex. That is, SIL_*C,n*_(*t*) = (*R*_1_, …, *R*_*k*_, *v*_1_, …, *v*_*k*_), where each *R*_*i*_ is a simplex, and *v*_*i*_ is the weight of that simplex, and the following are satisfied:

1. ${⋃}_{i=1}^{k}\bar{R_{i}}$ is a homogenous simplicial complex, where $\bar{R_{i}}$ is the simplicial complex consisting of *R*_*i*_ and all of its faces; and
2. The union of the simplices, ${⋃}_{i=1}^{k}R_{i}$, is convex.

As before, the silhouette is built recursively by expanding it over time to include motor trajectories that have been successfully used to communicate a selected concept (see Figure 2). But now that the regions specified by a silhouette are weighted, new motor trajectories will either add weight to the regions that it passes through (see Case 1) or it will affect the overall shape of the silhouette (see Case 2). The two cases are briefly described here.

**Figure 5:**
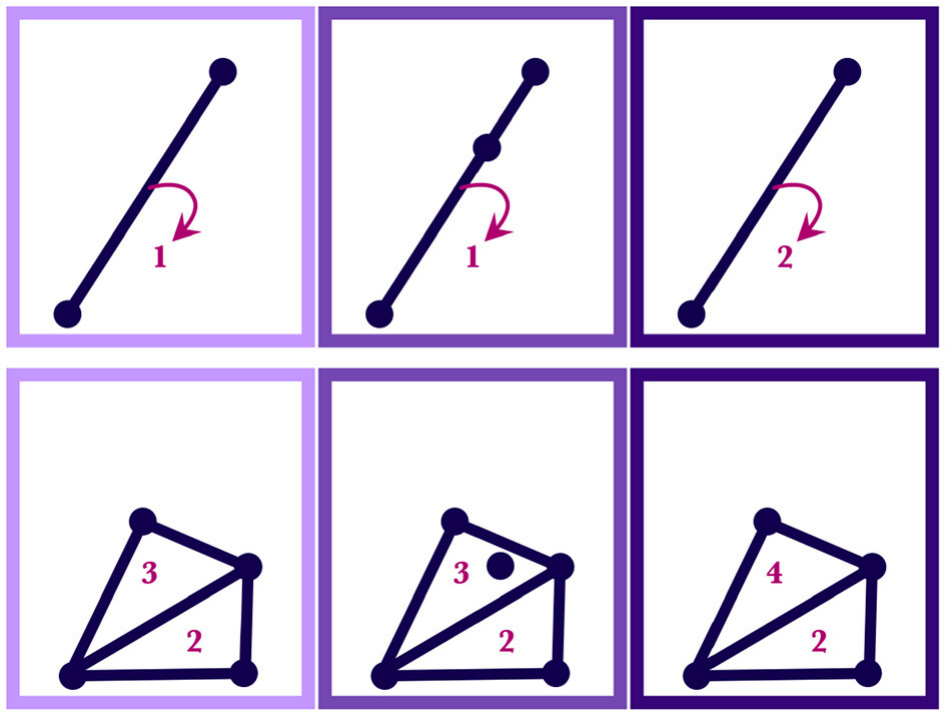
Both the upper and lower diagrams show how regions of a silhouette are reweighted as motor traces are absorbed by the wordform. In a given row, the leftmost panel shows the initial weighted region, SIL_*C,n*_(*t*); the middle panel shows the point, *M*(*t*), that will be added; the rightmost panel shows the resulting region, SIL_*C,n*+1_(*t*), with new weights. The numbers indicate the weights of the simplices. The upper diagram shows a simplicial 1-complex and the lower diagram shows a simplicial 2-complex.

Assume the speaker uses SIL_*C,n*_ to successfully communicate *C* using the motor trajectory *M*. Then the next iteration of the silhouette, SIL_*C,n*+1_, will be defined at time *t* in the following way:

**Case 1**. If *M*(*t*) is a point that is already in one of the simplices in SIL_*C,n*_(*t*), then SIL_*C,n*+1_(*t*) is the same as SIL_*C,n*_(*t*) except with the weight of the simplex (subregion) containing *M*(*t*) increased by one. Similarly, if *M*(*t*) is contained in multiple simplices—that is, if it lies on a shared boundary—then SIL_*C,n*+1_(*t*) is the same as SIL_*C,n*_(*t*) but with all the simplices containing *M*(*t*) having their weight increased by one. This case is illustrated in Figure 5.

**Figure 6:**
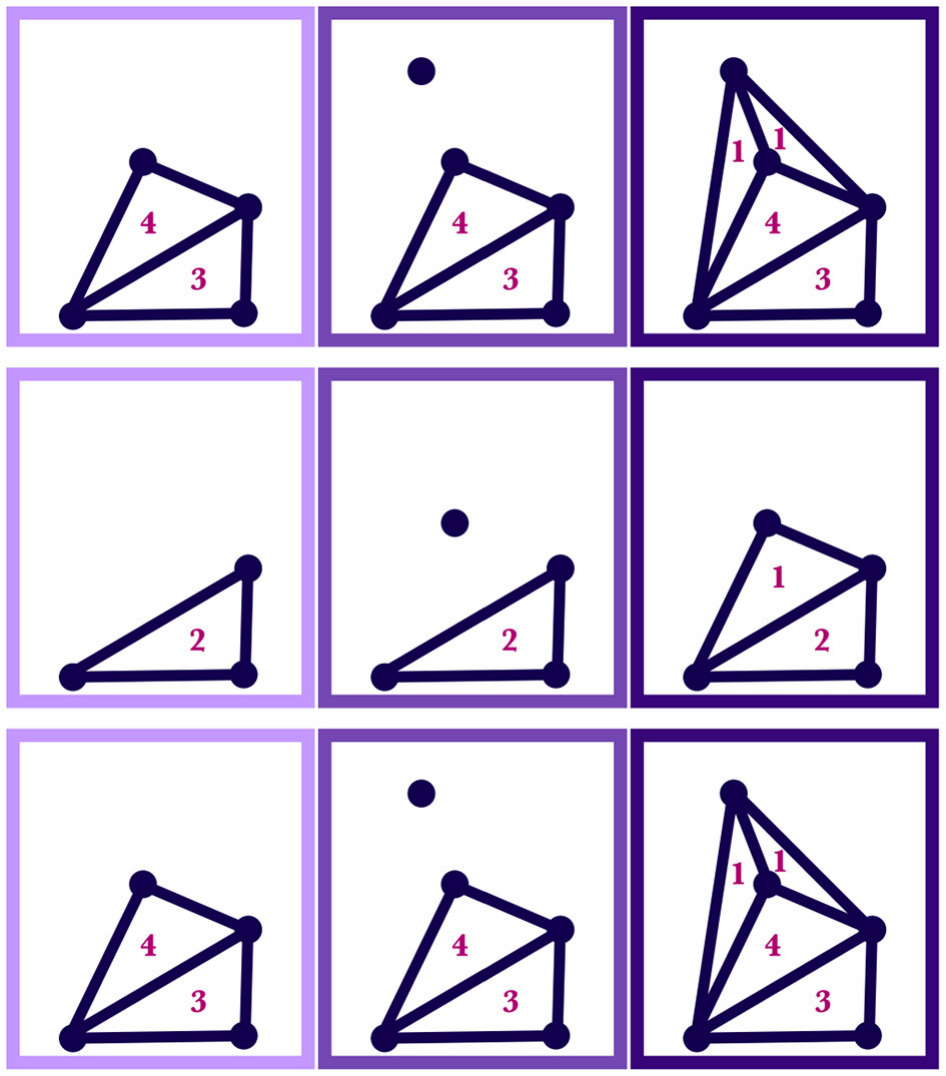
Each row of panels shows how a silhouette region, SIL_*C,n*_(*t*), changes with the inclusion of an additional point, *M*(*t*). In a given row, the leftmost panel shows the initial region, SIL_*C,n*_(*t*), with the numbers indicating the weights; the middle panel shows the point to be added, *M*(*t*); the rightmost panel shows the new resulting shape and weighting of the region, SIL_*C,n*+1_(*t*).

**Case 2**. On the other hand, if *M*(*t*) is totally outside SIL_*C,n*_(*t*), then SIL_*C,n*+1_(*t*) is created by adding a minimal number of simplices to SIL_*C,n*_(*t*) to create a homogenous simplicial complex in which *M*(*t*) is now contained, with the weights of the new simplices being 1. Examples of this case are illustrated in Figure 6.

The integration of a weighted silhouette, SIL_*C*_, and an exemplar, *e*_*C*_, will be similar to the integration described in the previous section but must take into account the weighting. The only thing that changes is how we compute the motor activation of a juncture. Suppose SIL_*C*_(*t*) = (*R*_1_, …, *R*_*k*_, *v*_1_, …, *v*_*k*_). Then we define the weighted motor activation of JUNCTURE_*i, j*_ to be the weighted average of the activations that come from each region:


$$\begin{array}{l} \text{M}\text{OTOR}\text{A}{\text{CTIVATION}}_{t}(\text{J}{\text{UNCTURE}}_{i,j})=\frac{1}{\sum_{s=1}^{k}v_{s}}\times \sum_{s=1}^{k}v_{s}\\ \;\times (\text{H}\text{IGHEST}\text{A}\text{CTIVATION}\text{M}\text{OTOR}\\ \;-(\text{D}\text{ROP}\text{O}\text{FF}\text{S}\text{LOPE}\text{M}\text{OTOR}\\ \;\times (\text{D}\text{ISTANCE}(R_{s},\text{J}{\text{UNCTURE}}_{i,j})))) \end{array}$$

#### The effect of practice on accuracy

**Figure 7:**
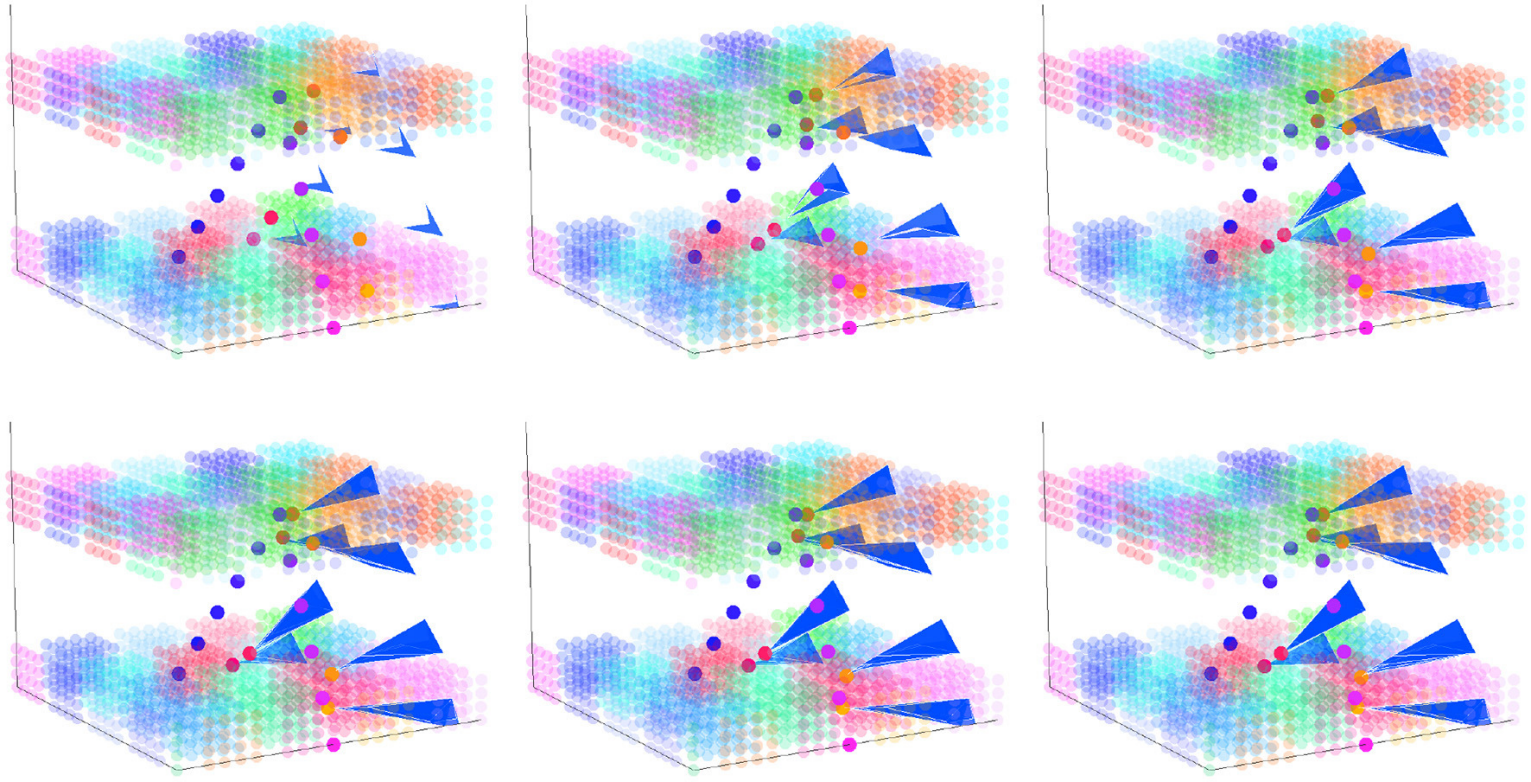
Silhouette change over time with each iteration of practice in a 3D perceptual-motor space where the perceptual and motor spaces have identical layouts. The upper-left panel shows the starting silhouette (blue triangular shapes), the exemplar trajectory (blue to pink dots), and the output trajectory (red to orange dots). Reading from right-to-left and then top-to-bottom, the figure illustrates how the silhouette changes in shape as it incorporates the output trajectory from each prior production.

To examine the effect of practice on learning and change in the model, we can use the silhouette at iteration *n* to produce an output trajectory that is absorbed as a motor trace into the silhouette; the new silhouette is then used for production at iteration *n* + 1. When we do this repeatedly (= practice), learning occurs with changes to the silhouette. Figure 7 shows what this change looks like, step-by-step, in a 3-dimensional space. The space represents the topology of clusters in both motor and perceptual space since these were identical in the simulation to facilitate the visualization of silhouette movement toward the exemplar in perceptual-motor space.

Imagine that the *z*-axis in Figure 7 represents a close–open vocal tract dimension in motor space and the aperiodic–periodic sound dimension in perceptual space, which do roughly correspond to one another. This would mean that activation of clusters near the *x*−*y* plane would result in consonantal-like articulations and that activation of clusters that are further above the *x* − *y* plane would result in vowel-like articulations. The silhouette, exemplar, and output paths in Figure 7 all travel from clusters near the *x* − *y* plane toward those furthest from this plane and then back again—a path that describes a CVC-shaped word. The upper-left panel shows a starting silhouette (blue triangular shapes) that might be an early representation of this word in that it is both far away from the exemplar trajectory (blue to pink dots) and is itself built up from only a few motor trajectories. With each of the 6 iterations of practice shown, the silhouette's path expands and changes shape: its weight gets distributed more toward the exemplar.

**Figure 8:**
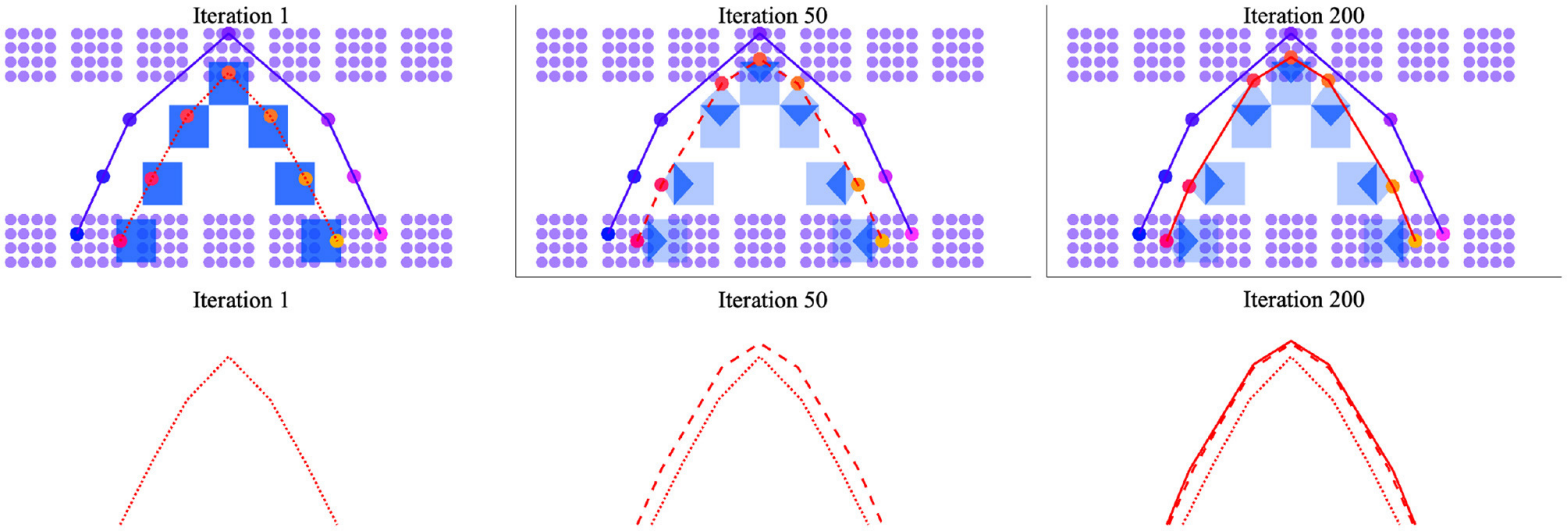
Change in output trajectory over time with iterations of practice in a 2D perceptual-motor space where the perceptual and motor spaces have identical layouts. **(Upper)** Silhouettes are shown as blue squares **(left)** or blue polygons of varying-opacity **(center and right)** to indicate weighting; the exemplar trajectory is traced in purple; the output trajectory in red. **(Lower)** The output trajectory is depicted after 1 iteration (dotted line), 50 iterations (dashed line), and 200 iterations (solid line). Reading from **(left-to-right)**, the output trajectory is shown to change shape to better approximate the exemplar trajectory over time.

Practice-based changes to the silhouette mean that, with time, the output trajectory will draw nearer to those clusters that are especially activated by the exemplar. This effect of practice is more easily visualized in 2-dimensional space than in 3-dimensional space. Figure 8 therefore displays the results of a simulation in 2D space where, similar to Figure 7, clusters are separated to model vowel- vs. consonant-like articulations and the motor and perceptual spaces have identical layouts. With this in mind, the exemplar trajectory shown in purple in the figure again describes a CVC trajectory. The silhouette in blue highlights a path that diverges from this trajectory. The output trajectory, which is linearly interpolated in red, is shown as a dotted line after the first time the exemplar and silhouette are integrated; it is shown as a dashed line after 50 iterations of the simulation and as a solid line after 200 iterations. Overall, the figure illustrates the expansion of the output trajectory in the direction of the larger exemplar trajectory with changes to the silhouette resulting from speech practice.

Intriguingly, the simulation result shown in Figure 8 indicates a period of relatively rapid change in production followed by a longer period of very marginal change. This unanticipated result is qualitatively similar to well-described patterns of early gains followed by plateaus in the motor learning literature (Adams, 1987; Newell et al., 2001). It also suggests that unsupervised speech practice is unlikely to drive substantial changes to production after a certain point. This is probably a good thing. After all, the persistent effect of “accent” in highly-proficient second language speakers would be hard to account for in the model if sheer practice were sufficient for a speaker to match exogenously-derived exemplars. Still, the result also suggests that other mechanisms besides practice are needed to describe the steep and relatively prolonged increase in speech production accuracy that is observed during the first 3 years of childhood. One possibility, not modeled here, is that feedback from listeners shapes learning— especially in children's speech when utterances are too short to present much in the way of context for the listener. This possibility is already an assumption of the overarching theory. Recall, that motor traces are only absorbed into the silhouette if communication is successful (Redford, 2019). Another possibility is that the production process can be perturbed to facilitate learning in such a way that merits, say, a return to the (slow) matching and selection process. If the speaker returns to the process of finding best perceptual matches between established motor trajectories and novel exemplars, new junctures may be created where different established trajectories near each other in motor space. The creation of new junctures may change the shape of existing clusters or establish new ones, thus changing the overall the structure of the perceptual-motor map in the direction of new ambient language input. Alternatively, the speaker may focus on the acoustic-perceptual shape of the word resulting in the up-weighting of contributions from the exemplar to overall cluster activation patterns during the integration process with consequences for the shape of the output trajectory. The theory allows for all of these alternatives.

### Novel word production

Although it is necessary to account for changes to known word production in a developmentally sensitive theory of production, it is not sufficient. This is especially true under the assumption of whole-word production as this assumption begets the problem of novel word production. Since we hypothesize that the default production strategy is silhouette–exemplar integration once an expressive vocabulary is established, the CC model adopts a silhouette-based approach to novel word production. Although the approach is motivated by the model architecture, it also allows us to capture an empirical finding from the literature on nonword repetition: the effect of vocabulary size on production accuracy in children's speech and in adult second language speech.

Not surprisingly, older children repeat nonwords more accurately than younger children and adults with more exposure to a second language repeat nonwords in the target language more accurately than those with less exposure. But accuracy also varies independently from age and experience with vocabulary size: children with smaller vocabularies repeat nonwords less accurately than children with larger vocabularies (e.g., Metsala, 1999; Verhagen et al., 2022); college-aged adults with smaller second language vocabularies produce less native-like renditions of nonwords than those with larger vocabularies (Bundgaard-Nielsen et al., 2012). Importantly, it is a child's expressive vocabulary size that correlates with production accuracy; not their overall vocabulary size (Edwards et al., 2004; Munson et al., 2005). In addition to vocabulary size, the production accuracy of novel words, or nonwords, varies with properties of the given nonword, including its “wordlikeness” and the relative frequency of its phonological patterning (e.g., Edwards et al., 2004; Guion et al., 2004; Munson et al., 2005; Redford and Oh, 2016). In brief, nonwords that obey the phonotactics of the (target) language and/or contain high frequency phonotactic patterns are repeated more accurately than those that are less “wordlike” with respect to phonotactics and/or contain less frequent patterns. The latter findings suggest that nonword production relies on existing wordform representations (Edwards et al., 2004; Guion et al., 2004; Redford and Oh, 2016).^4^ The CC model implements this hypothesis. When there is no silhouette for a given word, the speaker leverages the silhouettes that do exist to generate an archi-silhouette, or an A-silhouette, to provide the time-varying information needed to guide production. The A-silhouette is built by pulling together silhouettes from the nearest phonological neighbors of the targeted novel word form. In the psycholinguistic literature, phonological neighbors are wordforms that differ from one another by one phoneme (Luce and Pisoni, 1998). In the CC model, they are based on similarity in perceptual space, which is defined using the distance metric on that space. The algorithm for building an A-silhouette is described next.

#### Building an A-silhouette

Recall that the CC model has a function that measures distances between points in perceptual space. Let *d*_PERC_ be a function that measures the distance between perceptual trajectories (see Davis and Redford, 2019). The function operates by (1) aligning trajectories in perceptual space so their endpoints line up, using linear interpolation if necessary to fill in points, so that every point in one trajectory corresponds to one in the other, (2) finding the distances between corresponding points, and then (3) taking the average of these distances.

Now, suppose the speaker is attempting a new word *W* with exemplar *E*. Let *k* be a parameter with a fixed value representing the number of similar words from which to build an A-silhouette for *W*. For each word *w*_*i*_ (*i* = 1, 2, 3, … ) in the expressive lexicon, let *e*_*i*_ be its corresponding exemplar and let SIL_*i*_ be its corresponding silhouette. Assume that the expressive words are already ordered by perceptual closeness to *W*; that is, *d*_PERC_(*w*_1_, *W*) ≤ *d*_PERC_(*w*_2_, *W*) ≤ *d*_PERC_(*w*_3_, *W*) ≤ …  Then *w*_1_, *w*_2_, …, *w*_*k*_ are the *k* perceptually closest words to *W* in the expressive lexicon, and their silhouettes, SIL_1_,SIL_2_, …,SIL_*k*_, are chosen to build the A-silhouette.

We assume that the chosen silhouettes have already been modified so that they are aligned with each other in time. The A-silhouette is a silhouette ASIL such that at each time *t*, ASIL is defined as a combination of SIL_*i*_(*t*) for *i* = 1, 2, …, *k*. More specifically, fix *t* and let SIL_*i*_(*t*) = (*R*_*i*,1_, *R*_*i*,2_, …, *R*_*i*,*ni*_, *v*_*i*,1_, *v*_*i*,2_, …, *v*_*i*,*ni*_) where *R*_*i*, 1_, *R*_*i*, 2_, …, *R*_*i*,*ni*_ are the *n*_*i*_ subregions making up SIL_*i*_(*t*) and *v*_*i*, 1_, *v*_*i*, 2_, …, *v*_*i*,*ni*_ are their respective weights. The weights are scaled so that the maximum weight at time *t* is the same for each silhouette. That is, let MAXWEIGHT_*i*_ = max(_*v*_*i, j*_)*j* = 1, 2, …, *n*_*i*__, meaning MAXWEIGHT_*i*_ is the maximum weight of the regions in the *i*th silhouette (at time *t*). Then we use $v_{i,j}^{\prime }$ to denote the scaled version of *v*_*i, j*_, and we define $v_{i,j}^{\prime }=\frac{v_{i,j}\times \max{\left({\text{M}\text{AX}\text{W}\text{EIGHT}}_{i}\right)}_{i=1,2,\ldots ,k}}{{\text{M}\text{AX}\text{W}\text{EIGHT}}_{i}}$. That is, for each region, we take the original weight, multiply it by the maximum weight of all the regions in all the silhouettes, and then divide that by the maximum weight of the regions in that silhouette. Finally, the regions from all the silhouettes at time *t* are combined using the new weights. The combination process is demonstrated first with an example. The general process is given afterwards.

**Figure 9:**
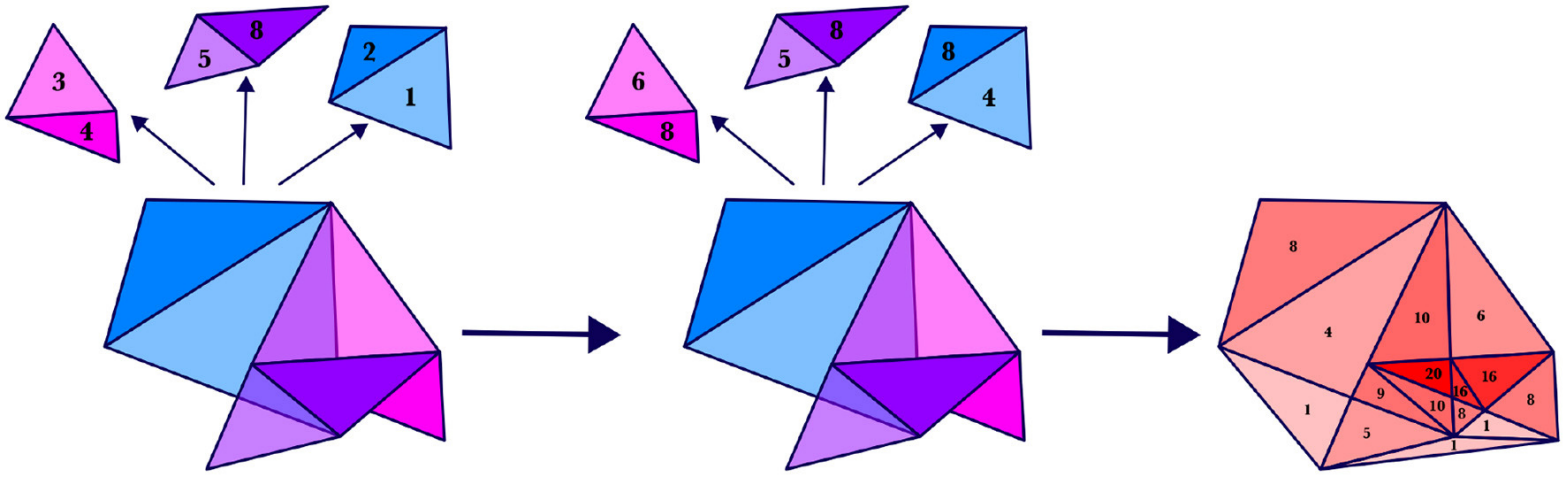
**(Left)** Regions corresponding to three motor silhouettes at a particular time; each silhouette at this time has a two subregions, with weights labeled. **(Center)** The regions with scaled weights. **(Right)** The weighted combination of the regions.


Suppose we have 3 aligned silhouettes, SIL_1_,SIL_2_, SIL_3_, and suppose that at time 2, each silhouette consists of two regions, *R*_1,1_ and *R*_1,2_; *R*_2,1_ and *R*_2,2_; and *R*_3,1_ and *R*_3,2_, respectively, where they overlap as shown in Figure 9. Suppose these regions have respective weights *v*_1,1_ = 3 and *v*_1,2_ = 4; *v*_2,1_ = 5 and *v*_2,2_ = 8; and *v*_3,1_ = 2 and *v*_3,2_ = 1. That is,


$$\begin{array}{ll} {\text{SIL}}_{1}\left(2\right) & =\left(R_{1,1},R_{1,2},3,4\right)\;\text{where }R_{1,1}\text{ and }R_{1,2}\text{ are the pink triangles}\\ {\text{SIL}}_{2}\left(2\right) & =\left(R_{2,1},R_{2,2},5,8\right)\;\text{where }R_{2,1}\text{ and }R_{2,2}\text{ are the purple triangles}\\ {\text{SIL}}_{3}\left(2\right) & =\left(R_{3,1},R_{3,2},2,1\right)\;\text{where }R_{3,1}\text{ and }R_{3,2}\text{ are the blue triangles} \end{array}$$

Then scaling the weights as described above yields a maximum weight of 8 for each region; that is,

$$\begin{array}{l} v_{1,1}^{\prime }=6,\;v_{1,2}^{\prime }=8,\;v_{2,1}^{\prime }=5,\;v_{2,2}^{\prime }=8,\;v_{3,1}^{\prime }=8,\;v_{3,2}^{\prime }=4. \end{array}$$

Then we will define the combination of these regions, ASIL(2), to be the weighted region shown in red. That is, ASIL(2) = (*T*_1_, *T*_2_, *T*_3_, *T*_4_, *T*_5_, *T*_6_, *T*_7_, *T*_8_, *T*_9_, *T*_10_, *T*_11_, *T*_12_, *T*_13_, *T*_14_, *T*_15_, 8, 4, 1, 10, 20, 10, 9, 5, 6, 16, 16, 8, 1, 8, 1), where *T*_*i*_ are the red triangles shown in Figure 9.

Returning to the general case where the selected silhouettes are SIL_1_, SIL_2_, …, SIL_*k*_, we define ASIL(*t*) = (*T*_1_, *T*_2_, …, *T*_*n*_, *v*_1_, *v*_2_, …, *v*_*n*_) where *T*_1_, *T*_2_, …, *T*_*n*_ is a triangulation of the convex hull of all the regions making up all the SIL_*i*_(*t*). For each *i*, the weight *v*_*i*_ of the region *T*_*i*_ is defined as follows: either (1) *v*_*i*_ is equal to the sum of the weights of all the original regions that *T*_*i*_ lies inside, or (2) *v*_*i*_ = 1 if it lies in none of the original regions but is still part of the convex hull.

That is, ASIL(*t*) = (*T*_1_, *T*_2_, …, *T*_*n*_, *v*_1_, *v*_2_, …, *v*_*n*_) such that

1. *T*_1_∪*T*_2_∪⋯∪*T*_*n*_ =CONVHULL(*R*_1,1_, *R*_1,2_, …, *R*_1,*n*_1__, *R*_2,1_, *R*_2,2_, …, *R*_2,*n*_2__, …, *R*_*k*, 1_, *R*_*k*, 2_, …, *R*_*k*,*nk*_)
2. Each *T*_*i*_ is a simplex (an “*n*-dimensional triangle")
3. The regions do not overlap each other more than at a boundary: interior(*T*_*i*_)∩interior(*T*_*j*_) = ⊘ for all 1 ≤ *i* < *j* ≤ *n*
4. For every set *A* = {*R*_*i*_1_, *j*_1__, …, *R*_*i*_*m*_, *j*_*m*__}, either ⋂_*a*∈*A*_*a* = ⊘ or ⋂_*a*∈*A*_*a* = *T*_*k*_1__∪*T*_*k*_2__∪⋯∪*T*_*k*_*s*__ for some *k*_1_, *k*_2_, …, *k*_*s*_ ∈ {1, 2, …, *n*}
5.
$v_{\ell }=\{\begin{array}{cc} \sum_{R_{i,j}\text{ containing }T_{\ell }}v_{i,j}^{\prime } & \text{if at least one }R_{i,j}\text{ contains }T_{\ell }\text{, i.e. if this}\\ 1 & \begin{array}{c} \text{sum is nonzero}\\ \text{ otherwise} \end{array} \end{array}$

#### The effect of vocabulary size on accuracy

According to the process outlined above, exemplars of words that belong only to the receptive vocabulary are attempted by combining the silhouettes of perceptually similar words that belong to the expressive vocabulary. But how good is this combined form? To what extent will it allow for a path through motor space that overlaps with the clusters activated by the novel exemplar in perceptual space? In this section, we demonstrate that the answer to these questions depends on the size of the expressive vocabulary. More specifically, we show that the goodness of the A-silhouette depends on the goodness of the perceptual matches to the novel wordform. The goodness of the perceptual matches in turn depends on the size of the speaker's expressive vocabulary, *V*, in relation to the larger vocabulary, *L*.

The larger vocabulary, *L*, is a theoretic construct that represents the set of words in a language over which the phonology is defined. The size of *L* depends on what exactly it represents. *L* could represent the size of a dictionary vocabulary or the size of an adult's overall vocabulary (10,000 words to 200,000 words) or the expressive vocabulary only, that is, half of the overall vocabulary size (Brysbaert et al., 2016). Alternatively, *L* could represent the total number of words required for normal every-day communication. We estimate that number here as 2500 words. This number is based on Nation and Waring's (1997) synthesis of research findings on the relationship between vocabulary size and second language acquisition for pedagogical purposes. Nation and Waring suggest that “a vocabulary size of 2,000–3,000 words provides a very good basis for language use.” This suggestion is based on the vocabulary size needed to achieve over 90% coverage of English texts aimed at young adult readers (e.g., 2,600 words result in 96% text coverage and a density of 1 unknown word occurring every 25 words). Insofar as young adults are perfectly good speakers of their native language, a vocabulary of roughly 2500 wordforms should adequately cover the phonological space of a language. It therefore provides a good basis for *L*.

Given that the words in *L* describe the phonological space for a particular language, it is clear that a subset *V* of *L* may fail to do so. And, if it fails to do so, then the A-silhouettes that are built up from wordforms in *V* are unlikely to reliably provide accurate information regarding the best path to take through motor space in order to approximate an exemplar that represents a novel word target. In particular, suppose *W* is the novel word, and suppose the A-silhouette is going to be built from the *k* words in *V* that are perceptually closest to *W*. What is the probability that these *k* words from *V* are actually some of the closest words to *W* in all of *L*? To make it more concrete, let *k* = 3 and let “best" be a synonym for “perceptually closest to *W*.” We can ask:

- What is the probability that the 3 best words in *L* are contained in *V* (and thus are also the 3 best words in *V*)?
- What is the probability that 3 of the 4 best words in *L* are contained in *V*?
- What is the probability that 3 of the 5 best words in *L* are contained in *V*?

More generally:

- What is the probability that 3 of the 3 + *r* best words in *L* are contained in *V*?

And even more generally:

- What is the probability that *k* of the *k* + *r* best words in *L* are contained in *V*?

**Figure 10:**
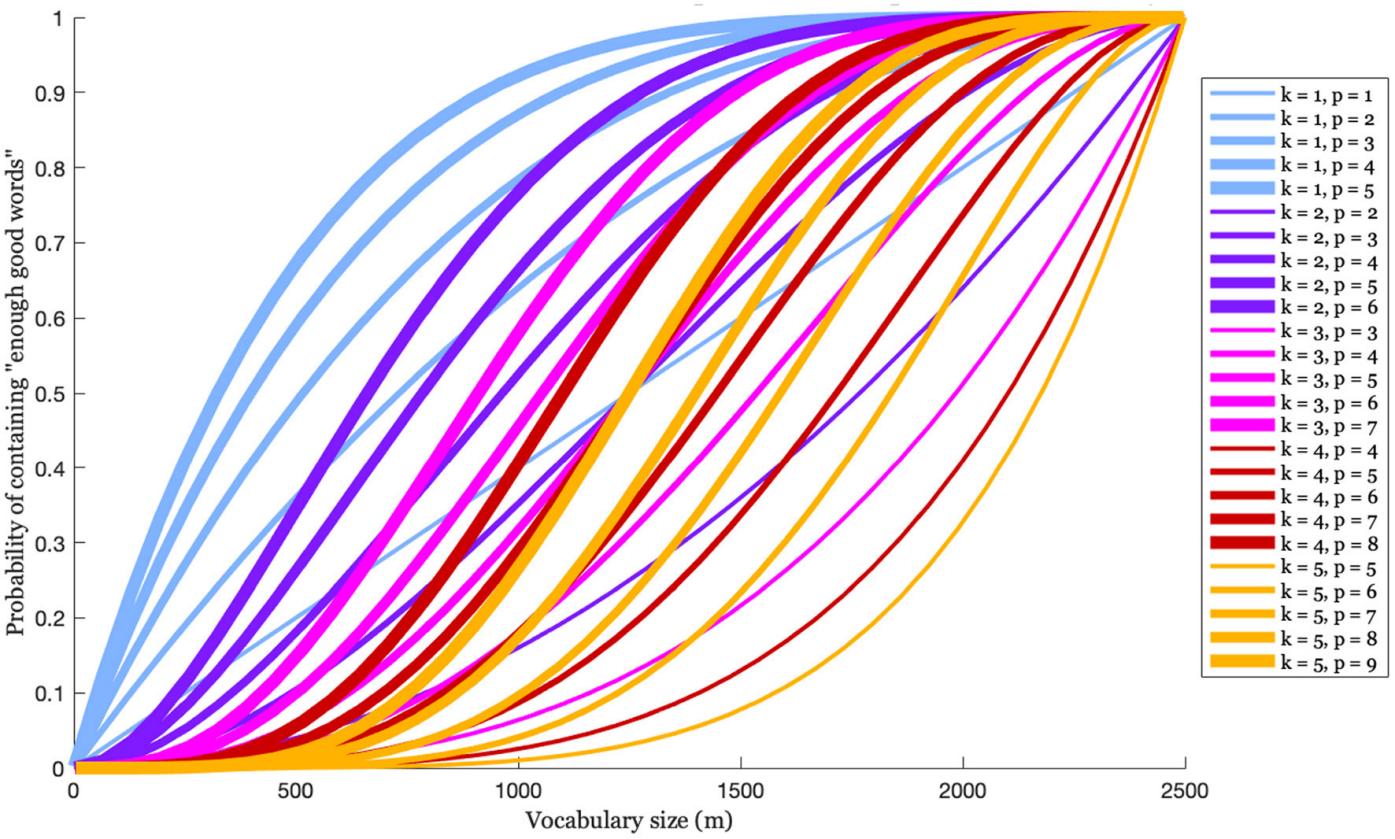
The probability that an expressive vocabulary of size *m*, drawn randomly from a larger vocabulary size of 2,500 words, contains at least *k* of the *p* words in *L* that are the closest perceptual matches to an exemplar that represents some novel word.

Naturally, this probability increases as the size of *V* increases. In particular, if *n* is the number of words in *L* and *m* is the number of words in *V*, the probability that *k* of the *k* + *r* best words in *L* are contained in *V*, i.e. that the *k* best words in *V* are a subset of the *k* + *r* best words in all of *L*, is:


$$\overset{r}{\underset{i=0}{\sum }}\frac{\left(k+r\right)!}{\left(k+i\right)!\left(r-i\right)!}\times \frac{\left(n-k-r\right)!}{\left(m-k-i\right)!\left(n-r-m+i\right)!}\times \frac{m!\left(n-m\right)!}{n!}$$

(assuming *k* ≤ *m* and *k* + *r* ≤ *n*). This is illustrated in Figure 10 for an *L* of size 2,500, and various values of *k* and *p*(= *k* + *r*). As the size of *V* increases (as we move right on the *x*-axis) the probability that the speaker's expressive vocabulary includes enough of the larger vocabulary's perceptually closest words to *W* also increases. This increase differs somewhat depending on the value of *k*, which, recall, is the number of words that are chosen to create the A-silhouette, and the value of *p*(= *k* + *r*), which is the number of words in *L* that are perceptually “close enough" to the novel word that any subset of *k* of those words could be used to create a very good A-silhouette for guiding production. The data here suggest that if *V* is 500—which is approximately the size of a typically-developing 3-year-old's expressive vocabulary (Shipley and McAfee, 2019)^5^—then it has a good chance (about 70%) of containing at least 1 of the 5 best words in *L*, but a poor chance (about 15%) of containing at least 3 of the 7 best words. This observation begs the question of how many closest perceptual wordforms are needed to generate an A-silhouette that will yield a good approximation of the novel word target. The data in the figure suggests that if in general any 3 of the closest 6 words to a goal word will yield a good A-silhouette, then good A-silhouettes can be reliably generated when *V* is 70% of *L*, or 1,750 words.

The predicted effect of an A-silhouette that is built up from a subset of “close enough” silhouettes is an output trajectory that approximates the exemplar of the novel word that is being attempted. Less good A-silhouettes result in less accurate output trajectories. To test this prediction, and so the effect of vocabulary size on the production accuracy, we simulated novel CVCV word production given different expressive vocabulary sizes and an all-CVCV language of 1,296 words. The language was built up from paths through a 2D motor space and a 2D perceptual space. The spaces had 6 clusters deemed consonantal articulations and 6 clusters deemed vocalic articulations. These groups of 6 were separated from one another in the *y* direction in motor space. The transformation from motor space to perceptual space was one that maintained this consonant-vowel separation, but shuffled the clusters in the *x* direction to render different topologies for the two spaces.^6^ The 1,296 wordforms were all the possible paths going from center-of-cluster to center-of-cluster in a CVCV-like pattern (1, 296 = 6 consonants × 6 vowels × 6 consonants × 6 vowels). The silhouettes consisted of 7 uniformly-weighted square regions, with regions 1, 3, 5, 7 centered on the appropriate CVCV clusters, and regions 2, 4, 6 falling evenly between them. The exemplar paired with a silhouette was built by taking the motor trajectory going through the center of the silhouette and finding the corresponding perceptual trajectory based on the transformation between the spaces.

In the simulation, the novel word was an exemplar randomly selected from the language. The initial expressive vocabulary consisted of 5 silhouette–exemplar pairs randomly selected from the 1,296-word language (minus the novel word). An A-silhouette was built from the 3 words in the expressive vocabulary that were perceptually closest to the novel word. An output trajectory was computed based on the integration of the A-silhouette and the novel word exemplar. The distance in perceptual space between the output trajectory and the novel word exemplar trajectory was calculated to measure the accuracy of the output trajectory. The initial vocabulary was then increased to 10 words by adding an additional 5 random CVCV words to the expressive vocabulary. A new A-silhouette was made, again using the 3 closest words, an output trajectory computed, and the distance in space from the exemplar calculated. The expressive vocabulary was next increased to 20, then 40, and so on for a range of sizes up to 1,200. For each vocabulary size, the output trajectory based on A-silhouette–exemplar integration was found and the distance from the novel word exemplar calculated.

**Figure 11:**
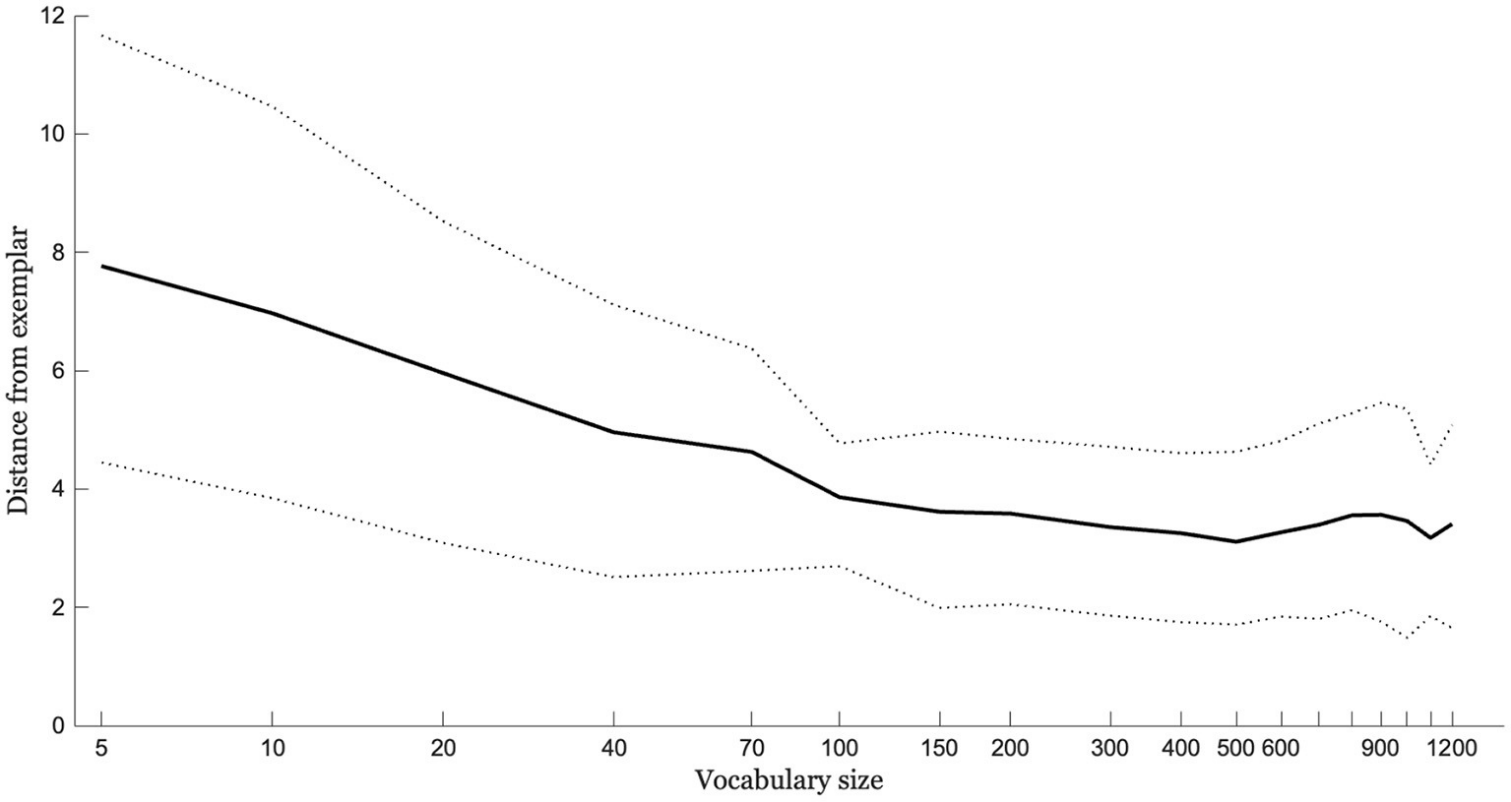
The average distance of the output trajectory from a novel word exemplar given integration based on an A-silhouette built from the 3 perceptually-closest silhouettes to the novel word exemplar. Distances are shown as a function of vocabulary size (log-transformed). Dotted lines show ±1 standard deviation around the mean for the simulation, which was run 20 times.

The entire simulation was run 20 times with different randomly-selected novel words and expressive vocabularies. Figure 11 shows the mean distance between output and exemplar trajectory as a function of vocabulary size for the 20 runs. The data indicate increasing production accuracy with increasing vocabulary size. The increase is steeper early on and more gradual later on. The pattern qualitatively matches the very robust increases in production accuracy seen during the earliest stages of speech acquisition followed by slower gains but continuing improvement.

## Summary and conclusion

The CC model captures the observation that speech develops with language use to address the problem of learning and change in production. The child's first words represent both a first attempt at speech and a first attempt to communicate using language. Control over speech action evolves in this communicative context with speech practice. And we engage in a whole lot of practice. The estimate from voice recordings of college-aged adults is that we speak about 16,000 words a day (Mehl et al., 2007). This kind of practice must have implications for speech production. In our theory it does.

The theory assumes a dual lexicon and whole-word speech production. The motor wordforms (silhouettes) in the lexicon are endogenous representations built up with speech practice. The perceptual wordforms (exemplars) are exogenous representations that reflect ambient language patterns. Speech production is the integration of these forms in the perceptual-motor map. The perceptual-motor map is discretized with vocal-motor practice, including speech practice, into language-specific clusters that represent units of speech motor control. The perceptual aspect of these units can be related to sound categories or to perceptual features; the motor aspect to vocal tract constrictions similar in some respects to the “gestures” of Articulatory Phonology except that do not necessarily code meaning contrast. They are units that represent both acoustic-auditory goals and spatial targets for the speech motor system.

When a word is selected for output from the expressive vocabulary, its silhouette and exemplar activate clusters in motor and perceptual space. The silhouette contributes time-varying information about movement through motor space within a window of activation that allows contextual effects to emerge (i.e., syntagmatic relations). The exemplar provides static information about the acoustic-auditory goals to be achieved for successful communication (i.e., paradigmatic relations). Perceptual-motor integration of the forms results in an output trajectory that traces speech movement due to the integration process. If the speech movement described by an output trajectory results in successful communication, then its trace is absorbed into the silhouette for the concept intended and communicated. By this mechanism, the silhouette for a word is shifted in the direction of the exemplar(s) of a word. This is the practice-based mechanism for motor learning and change in the model. Simulation results suggest that practice has a large initial effect on production accuracy, and that this effect plateaus relatively quickly, or is, at least reduced to only a very marginal effect over time. Overall, the pattern recalls the power law function of motor learning (see Newell et al., 2001).

Learning and change in the model also occurs with novel word production. In a system where silhouette–exemplar integration is the dominant mode of production, the accurate rendition of a novel word requires a silhouette-like form to achieve the targeted exemplar. The new silhouette, an A-silhouette, is created by combining existing silhouettes, which are selected based on the closeness of their perceptual counterparts to the novel-word exemplar. The algorithm for combining existing silhouettes to generate an A-silhouette relies on the model-internal fact that the expressive lexicon is structured according to the perceptual and motor spaces within which the dual wordforms reside. The receptive-only lexicon is also structured by the perceptual space within which single wordforms reside alongside their dual wordform neighbors. Although merely a logical consequence of the CC model architecture, the phonetically-structured lexicon of our theory parallels the well-established psycholinguistic hypothesis of a phonologically organized lexicon (Pisoni et al., 1985; Luce and Pisoni, 1998).

The integration of an A-silhouette and an exemplar associated with a novel word results in an output trajectory. The extent to which this output trajectory is similar to the exemplar varies naturally with vocabulary size. Smaller vocabularies do not regularly allow for the same quality of perceptual matches as larger vocabularies and so the A-silhouettes that are created based on a small vocabulary result in poorer production accuracy than those created based on larger vocabularies. This implication of the model is consistent with the effect of vocabulary size on nonword repetition accuracy in children's speech and in adult second language speech.

### Why core?

The CC model provides an intellectual framework within which to understand developmental changes in speech production. For this reason, it also provides a framework for understanding the emergence of individual differences in speech production, including differences due to developmental disorder. The model perspective is that these differences are the result of developmental trajectories that are themselves defined by iterative processes that may compound over time the effects of small differences in initial parameter settings.

No existing linguistic or psycholinguistic theory of speech production that we know of has been advanced with the particular aim of explaining change in a manner that naturally gives rise to different outcomes. To the best of our knowledge, every instantiated theory that handles adult spoken language production assumes (more or less) current descriptions of the adult speech behavior as its starting and ending point. They are teleological in this way. For this reason, individual differences are often treated as specific deviations from normativity rather than as the product of differing initial conditions and constraints on development. The teleological frame is, in part, the legacy of Saussure and his emphasis on the synchronic over the diachronic. It is, in part, the legacy of Chomsky and his emphasis on what is universal and so what might be innate. Collective knowledge about speech and language has grown enormously under these legacies. Our goal is to reframe some of this existing knowledge within an emergentist framework to better understand individual differences and to encourage new avenues of empirical research.

### Future directions

The Core/CC model framework emphasizes the role of variability in learning and change. Recall that speakers can only target previously experienced paths through motor space, even when attempting a new perceptual goal (sound or word). Under this hypothesis, noise in the periphery due to immature motor control provides an important learning benefit, not least of which is better and more thorough exploration of the motor space than would otherwise be possible; and it is through exploration that junctures proliferate in the perceptual-motor map in the first place. Clusters, the units of speech motor control, are created from these junctures. Clusters allow speakers to achieve language-specific acoustic-auditory goals. The proliferation of junctures in motor space is a prerequisite for doing so. The highly variable speech movements of children's speech compared to adults' speech may therefore be what allows them to acquire native-like speech sound articulation in a second language—something that adult learners are purportedly unable to do. The prediction is then for an increase in perceived accentedness in speech with age of acquisition, but one that tracks more specifically with age-related changes in the variability of speech movements. Age-of-acquisition effects are, of course, well-described in studies of second language speech—in fact, the age of 5–7 years has been suggested as a cut-off for nativelike acquisition of a second language speech category (e.g., Guion, 2003)–but the explanation for why this might be is elusive. Our prediction suggests that the cut-off is causally tied to the rapid leveling off of articulatory variability during developmnet (see, e.g., Smith and Zelaznik, 2004). Also, note that, just as children's speech continues to exhibit greater variability than adult speech until age 12–14 years, so too the age-of-acquisition effect on second language speech is graded—there is not an abrupt cut-off in native-like attainment of a second language at age 5 or 7 years across all individuals. Future research on second language acquisition could investigate the extent to which greater variability in the realization of sounds at one stage in development predicts more accurate (= target-like) attainment of these sounds at a later stage.

The Core/CC model framework also predicts a relatively abrupt transition from a period of exceptionally high variability in the production of novel words to a period of relative stability in word production that corresponds to a change in strategy from the matching and selection of existing motor trajectories to create best perceptual approximations of novel exemplar trajectories to a strategy based on an expressive vocabulary and so on the integration of perceptual and motor wordforms. Consistent with this, Vihman (2014) describes a shift in word production around 2 years of age that she attributes to a shift away from a strategy of schema-based production and toward template-based word production. Our A-silhouettes might be considered templates in that they are not word-specific, but rather an amalgam of similar sounding words. Vihman (2014) also notes that a schema-based and templatic-based production strategy may co-exist for some time during development, and that some children never really exihibit a phase that can truly be described as templatic. The CC model suggests that the path toward understanding these individual differences is through more careful study of the relationship between expressive vocabulary and phonological development during the young preschool years. This study should include not just the size of the expressive vocabulary, but also its detail regarding its phonological structure in perceptual and motor spaces.

In the CC model, the extent to which A-silhouettes allow for matching exogenous wordform representations varies with the size and structure of the expressive vocabulary. As already noted, this pattern is consistent with the effect of vocabulary size on nonword repetition accuracy in children's speech. But a detailed consideration of this relationship leads us now to wonder about an inflection point in development when production is no longer driven by the integration of the specific perceptual and motor wordforms that are stored together in an expressive vocabulary. Rather, it could be driven by the integration of perceptual wordforms and A-silhouettes. What this might mean is a question for future research. But, to give that research some structure, let us consider the problem in a little more detail.

Under the simplifying assumption that an expressive vocabulary is some random subset of the words in a language, it is clear that an A-silhouette will provide as good guidance as a more specific motor wordform once the expressive vocabulary reaches a certain size. The question then becomes: What is that certain size? This depends in part on the number of words needed to adequately describe the language. In our simulations, the language vocabulary was 2,500 words. This number of words was chosen on the grounds that between 2,000 and 3,000 words is adequate for everyday communication in English. We presume that this means that a specific set of 2,500 words adequately describes the phonology of English. But the number 2,500 was also chosen with young children's speech patterns in mind. In particular, 30% of 2,500 words is 750 words, which is a good approximation of a 3-year-old's expressive vocabulary size. And, since we know that 3-year-old speech is different from adult speech, it was convenient to consider the potential shape of A-silhouettes in this context. But the reader will have also noted that 2,500 words falls well short of the average expressive vocabulary size of a typical adult. In fact, the lower bound estimate of an average adults' expressive vocabulary size is 10,000 words; and, 30% of 10,000 words is even larger than our language vocabulary estimate. Given this, by the logic of our own model, 10,000 distinct silhouettes are clearly not required to produce 10,000 words. This observation suggests several paths for future research, including a version of the prior suggestion: more careful studies of the structure and size of developing expressive vocabularies are needed to better understand the relationship between the accuracy with which a novel word can be produced and the size of the expressive vocabulary.

Finally, the developmental perspective adopted here motivates our view that perceptual experience and motor practice interact and build on each other through time; together, they provide the foundation for an individualized account of spoken language patterns. The Core/CC model framework assumes the evolution of speech perception and of perceptual wordform representations, but addresses only the effects of motor practice on change. This limitation argues for future research that has as its aim to understand, in precise terms, how much of developmental change in the sound patterns of speech is due to perception and how much is due to production. It will also be important to determine how exactly to tell the difference between the two. The Core model framework suggests, consistent with much other theory, that perceptually-driven changes should be in the direction of increasing contrasts, and that motor-driven changes are in the timing domain. But timing differences also give rise to contrast. This is, in fact, the foundational insight on which Articulatory Phonology was built (i.e., language-specific gestural coordination). So, again, under the now well-articulated assumption of a dual lexicon, future research will need to detail the separate and interacting contributions from perceptual learning and speech motor learning to understand the emergence and evolution of individualized speech patterns.

## Data availability statement

Publicly available data for this study can be found at: https://github.com/mayaekd/core.

## Author contributions

The research reported here was fully collaborative. Both authors contributed to the writing and approved the submitted version of the manuscript.

## Footnotes

^1^Time is modeled discretely for computational reasons, but the concept is one of a continuous unfolding process (see Davis and Redford, 2019).

^2^Style-shifting is not addressed in this paper, but can be modeled within CC as the greater weighting of either the motor or perceptual activation pattern during integration. A reviewer points out that style could also be modeled in other ways within the model, including by the selection of specific formal or casual exemplars of words or by changing the size of the look-back and look-ahead windows of integration. This is also true. The main point here is that the distinct motor and perceptual activation patterns in the CC model are meant to incorporate the tension between “ease” and “distinctiveness” that is at the heart of Lindblom's H&H theory of production.

^3^Recall that the silhouette incorporates motor traces of words that were successfully communicated. This allows for the influence of the periphery (i.e., articulation) on representation. The periphery introduces noise into the representation in any number of ways, including by virtue of poorly established “functional synergies” (see, e.g., Smith and Zelaznik, 2004).

^4^For a substantially different interpretation of these findings see Gathercole (2006).

^5^This assumes an expressive vocabulary that is half the size of the overall vocabulary, which Shipley and McAfee (2019) place at about 1,000 words for a typically-developing 3-year-old.

^6^Specifically, the clusters were 4 × 4 squares of 16 junctures, with the horizontal distance between two adjacent junctures within a cluster being 1 and the horizontal distance between two adjacent clusters being 2. The vertical distance between adjacent junctures within a cluster was 1 and the vertical distance between the bottom row of clusters and the top row of clusters was 15. Let us designate the bottom-row clusters as “consonants” and the top as “vowels.” The transformation between motor and perceptual space can then be described as follows: If in motor space, the consonants from left to right were *C*_1_, *C*_2_, *C*_3_, *C*_4_, *C*_5_, *C*_6_, then in perceptual space they were *C*_3_, *C*_4_, *C*_1_, *C*_2_, *C*_5_, *C*_6_; if in motor space, the vowels from left to right were *V*_1_, *V*_2_, *V*_3_, *V*_4_, *V*_5_, *V*_6_, then in perceptual space they were *V*_3_, *V*_4_, *V*_5_, *V*_6_, *V*_1_, *V*_2_.
